# Milk lactoperoxidase decreases ID1 and ID3 expression in human oral squamous cell carcinoma cell lines

**DOI:** 10.1038/s41598-020-62390-4

**Published:** 2020-04-03

**Authors:** Layla Panahipour, Maria De Biasi, Theresa Sophia Bokor, Alexandra Thajer, Nadja Haiden, Reinhard Gruber

**Affiliations:** 1 Department of Oral Biology, Medical University of Vienna, Sensengasse 2a, 1090 Vienna, Austria; 2 Department of Paediatrics and Adolescent Medicine, Medical University of Vienna, Währinger Gürtel 18-20, 1090 Vienna, Austria; 3 Department of Clinical Pharmacology, Medical University of Vienna, Währinger Gürtel 18-20, 1090 Vienna, Austria; 4 Department of Periodontology, School of Dental Medicine, University of Bern, Freiburgstrasse 7, 3010 Bern, Switzerland; 5 Austrian Cluster for Tissue Regeneration, Donaueschingenstraße 13, 1200 Vienna, Austria

**Keywords:** Oral cancer, Oral cancer

## Abstract

Milk consumption may modify the risk of squamous cell carcinoma. The role of milk to modulate the gene expression in oral squamous cell carcinoma cells has not been investigated so far. Here, HSC2 oral squamous carcinoma cells were exposed to an aqueous fraction of human milk and a whole-genome array was performed. Among the genes that were significantly reduced by human and cow milk were the DNA-binding protein inhibitor 1 (ID1), ID3 and Distal-Less Homeobox 2 (DLX2) in HSC2 cells. Also, in TR146 oral squamous carcinoma cells, there was a tendency towards a decreased gene expression. Upon size fractionation, lactoperoxidase but not lactoferrin and osteopontin was identified to reduce ID1 and ID3 in HSC2 cells. Dairy products and hypoallergenic infant formula failed to decrease the respective genes. These data suggest that milk can reduce the expression of transcription factors in oral squamous carcinoma cells.

## Introduction

There is a continuing debate of whether or not intake of milk is a risk factor in oral squamous cell carcinoma, aggressive cancer arising from a neoplastic dysplasia that progresses to malignancy^1^. Milk intake can lower the risk of tea drinkers for oral squamous cell carcinoma^2^. However, in the oral cavity and pharyngeal cancer, a positive association was found for milk and dairy products^3^. Milk and dairy products were associated with head and neck squamous cell carcinoma^4^. There is also a discrepancy with other tumor entities. Meta-analyses revealed a reduction of bladder cancer in milk consumers^5^, no effect on ovarian cancer^6^, and an increased risk for prostate cancer^7^. Furthermore, the possible risk of milk and dairy product consumption in gastric cancer was analyzed^8^. Even though epidemiological studies revealed associations of milk intake and the occurrence of tumors, the underlying molecular mechanisms of how milk and dairy products can affect squamous cell carcinomas have not been investigated so far.

Milk being produced by the mammary gland provides infants and small children with nutrients but also protects against necrotizing enterocolitis^9–11^. Moreover, the topical application of milk supports umbilical cord separation in newborns^12–15^ and the healing of diaper dermatitis^16^, ulcerated haemangioma^17^, and atopic eczema^18^. The role of milk and dairy products for oral health has not been investigated so far in a clinical setting. We have previously shown that milk holds an anti-inflammatory activity for cells of the oral cavity including gingival fibroblasts, macrophages but also the squamous cell carcinoma cell line HSC2^19^. Moreover, milk provokes a TGF-β response in gingival fibroblasts^20^. Milk is also a rich source of lactoperoxidase^21^, lactoferrin^22^ and osteopontin^23^, all causing cellular responses. These findings have prompted us to determine the reaction of oral squamous cell carcinoma cells to milk using molecular screening approaches. The overall concept was to identify genes in the oral squamous carcinoma cell being regulated by milk.

## Results

### Gene array of HSC2 cells exposed to human milk

**Figure 1:**
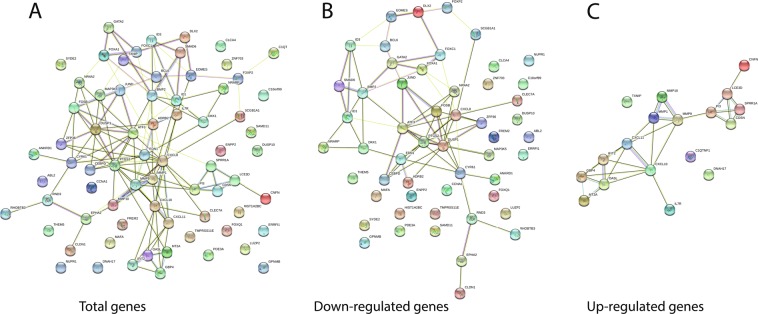
STRING analysis of milk-regulated genes in HSC2 cells. HSC2 cells when exposed to 5% aqueous fraction of pasteurized human milk express genes that were revealed by whole-genome gene array that can be clustered by STRING analysis.

To reveal the spectrum of strongly regulated genes, HSC2 cells were exposed to 5% of an aqueous fraction of pooled pasteurized human milk and a gene array was performed. Previous viability tests showed no changes in cell viability when cells were exposed to 10% aqueous fraction of human milk, cow milk and infant formula (Suppl. Fig. 1). We could identify 70 genes being at least 5-fold regulated that were subjected to gene ontology analysis (Fig. 1; Suppl. Table 1). The main Molecular Functions were all related to regulation of gene expression e.g. GO:0008134 (transcription factor binding), GO:0000977 (RNA polymerase II regulatory region sequence-specific DNA binding), GO:0044212 (transcription regulatory region DNA binding), GO:0001228 (DNA-binding transcription activator activity, RNA polymerase II-specific), and GO:0005515 (protein binding). In line with this category, also GO:0000122 (negative regulation of transcription by RNA polymerase II) was among the most relevant Biological Process regulated in HSC2 cells by milk. With respect to their role in oncology, it was particular GO:0000981 (DNA-binding transcription factor activity, RNA polymerase II-specific) that draws our attention to the genes ID1, ID3 and DLX2. In the category “InterPro” Protein Domains and Features, IPR026052 (DNA-binding protein inhibitor), as well as in KEGG, hsa04350 (TGF-beta signaling pathway) highlighted - both ID1 and ID3 to be regulated by milk (Suppl. Table 2).

### Gene expression analysis of HSC2 exposed to milk

**Figure 2:**
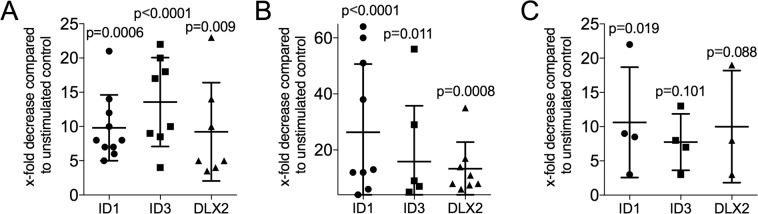
Decreased ID1, ID3 and DLX2 expression in HSC2 exposed to milk. HSC2 oral squamous cell carcinoma cells were exposed to 5% of aqueous fractions of pasteurized human milk (**A**), cow’s milk (**B**) and infant formula (**C**) for 24 hours, before expression analysis of the target genes ID1, ID3 and DLX2 were performed. Data indicate the x-fold decrease normalized to unstimulated control cells. The experiments performed at least three times.

To confirm the findings of the gene array approach, traditional RT-PCR on the target genes ID1, ID3 and DLX2 were performed. Exposure of HSC2 to 5% aqueous fraction of human milk caused a median 8.0, 13.5 and 5.0-fold decrease of ID1, ID3 and DLX2 (Fig. 2A). Also, cow’s milk (Fig. 2B) and infant formula (Fig. 2C) similarly decreased the expression of ID1, ID3 and DLX2 in HSC2 cells. Dose-response experiments revealed that cow milk and infant formula, and to a lower degree also human milk, require a minimum concentration of 0.5% to provoke changes of the three main target genes in HSC2 cells (Table 1). There was also a time-dependency of human and cow milk to lower ID1, ID3, and DLX2, starting at 30 minutes and lasts at least 24 hours (Table 2). We also tested a series of other genes that were identified with the gene array but were not considerably regulated by human milk in HSC2 cells based on RT-PCR (Supplement Table 3 with primer sequence). Gingival fibroblasts showed no considerable changes in the target genes in a similar setting (data not shown). Taken together, it was particular the HSC2 cells responding to milk with a strong decrease of ID1, ID3 and DLX2 expression.

**Table 1:** Dose-response of ID1, ID3 and DLX2 expression in HSC2 cells exposed to milk.

MM	5%	0.5%	0.05%
ID1	4.5 ± 1.5	1.5 ± 0.6	1.5 ± 0.6
ID3	7.3 ± 2.1	1.7 ± 0.6	1.5 ± 0.6
DLX2	5.9 ± 3.0	1.8 ± 0.5	2.2 ± 1.1
CM	5%	0.5%	0.05%
ID1	7.7 ± 1.9	2.4 ± 7.5	1.6 ± 0.3
ID3	6.5 ± 1.8	2.7 ± 0.8	1.7 ± 0.4
DLX2	1.9 ± 0.2	2.2 ± 0.5	1.4 ± 0.2
IF	5%	0.5%	0.05%
ID1	8.5 ± 2.9	1.8 ± 0.4	1.8 ± 0.2
ID3	14.3 ± 5.	62.4 ± 0.	41.8 ± 0.2
DLX2	6.5 ± 3.3	2.2 ± 0.6	1.8 ± 0.3

The oral squamous cell carcinoma cell line HSC2 was exposed to the indicated concentrations of aqueous fractions of pasteurized human milk (MM), cow’s milk (CM), and infant formula (IF) for 24 hours, before expression analysis of the target genes ID1, ID3 and DLX2 were performed. Data indicate the x-fold decrease compared to unstimulated control cells. Data represent three independent experiments.

**Table 2:** Time-response of ID1, ID3 and DLX2 expression in HSC2 cells exposed to milk.

MM	30 min	60 min	3 hours	24 hours
ID1	4.5 ± 3.9	3.6 ± 3.7	18.5 ± 22.3	11.5 ± 8.2
ID3	19.6 ± 21.6	17.7 ± 22.9	13.4 ± 8.4	16.3 ± 10.2
DLX2	2.6 ± 0.1	1.1 ± 0.8	34.8 ± 46.1	5.6 ± 1.5
CM	30 min	60 min	3 hours	24 hours
ID1	12.6 ± 14.7	35.9 ± 65.4	16.0 ± 19.7	30.9 ± 40.0
ID3	7.0 ± 7.6	19.2 ± 23.6	5.5 ± 3.4	24.1 ± 19.7
DLX2	3.5 ± 3.7	4.4 ± 3.9	16.3 ± 19.5	43.4 ± 71.1

The oral squamous cell carcinoma cell line HSC2 was exposed to 5% aqueous fractions of pasteurized human milk (MM), cow’s milk (CM), and infant formula (IF) for the indicated time period, before expression analysis of the target genes ID1, ID3 and DLX2 were performed. Data indicate the x-fold decrease compared to unstimulated control cells. Data represent three independent experiments.

### Gene expression analysis of HSC2 exposed to dairy products

Neither of the dairy products – yogurt, buttermilk, sour milk and whey - provoked considerable changes of ID1, ID3 and DLX2 expression (Suppl Fig. 2). In support of these observations, lowering the pH in pasteurized cow milk by adding 2% of lactic acid almost abolished the activity of the corresponding aqueous fraction (Suppl Fig. 3). Moreover, 0.2% of 2′-fucosyllactose (2′FL) and 3-fucosyllactose (3FL), both are milk oligosaccharide (HMOs), could not replace milk in lowering the expression of the gene panel (Suppl Fig. 4). Moreover, considering that milk is a rich source of activin A^24^, and activin A can decrease ID1 and ID3 expression in ovine granulosa cells^25^, we exposed HSC2 cells to recombinant activin A; however no considerably changes were observed in our *in vitro* setting (Suppl Fig. 5A). Correspondingly, an activin A neutralizing antibody failed to reverse the milk-induced decreased of ID1 and ID3 expression in HSC2 cells (Suppl Figs. 5B,C).

### Gene expression analysis of TR146 cells and gingival fibroblast exposed to milk

**Figure 3:**
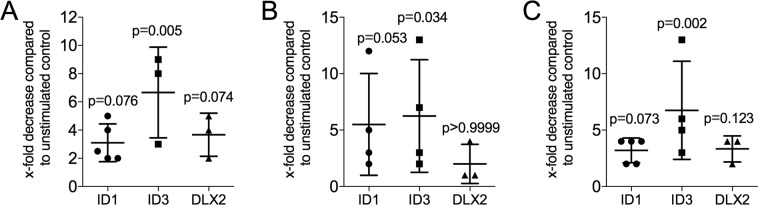
Decreased ID1, ID3 and DLX2 expression in TR146 exposed to milk. TR146 oral squamous cell carcinoma cells were exposed to 5% of aqueous fractions of pasteurized human milk (**A**), cow’s milk (**B**), and infant formula (**C**) for 24 hours, before expression analysis of the target genes ID1, ID3 and DLX2 were performed. Data indicate the x-fold decrease normalized to unstimulated control cells. The experiments performed at least three times.

Human milk, cow milk and infant formula (Fig. 3) caused a lowering of ID3 expression but did not reach the level of significance on ID1 and DLX2 in TR146 cells. Again, neither yogurt, buttermilk, sour milk or whey caused significant changes of ID1, ID3 and DLX2 expression in TR146 cells (Suppl Fig. 6). No changes in the respective target genes were obtained in gingival fibroblasts when incubated with human, cow milk and infant formula (data not shown).

### Protein expression analysis of HSC2 exposed to milk

**Figure 4:**
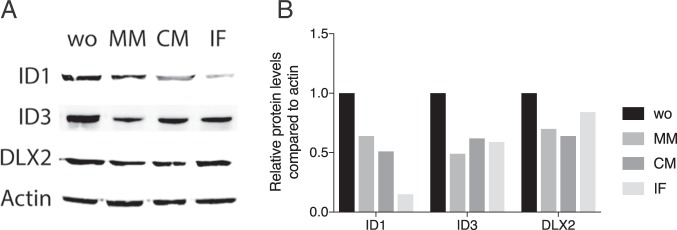
Western blot of ID1, ID3 and DLX2 in HSC2 exposed to milk. HSC2 oral squamous cell carcinoma cells were exposed to 5% of aqueous fractions of pasteurized human milk (MM), cow’s milk (CM), and infant formula (IF) for 72 hours. (**A**) Western blot analysis of ID1, ID3 and DLX2 was performed. (**B**) Data indicate the relative changes normalized to actin and unstimulated control cells.

We next investigated whether the findings obtained on the transcriptional level can translate into a protein. Western blot analysis revealed that after 72 hours of exposure of HSC2 cells, the aqueous fractions of human milk, cow milk and infant formula decreased the levels of ID1 and ID3 on the protein level. The changes in DLX2 on the protein level were not that obvious (Fig. 4A). The densitometric data are expressed in Supplementary Table 4 and in Fig. 4B. Thus, aqueous fraction of pasteurized human milk, cow milk and infant formula obviously lower the protein levels of ID1 and ID3 in HSC2 cells.

### Gene expression analysis of HSC2 exposed to lactoperoxidase

To identify at least one active component which may be responsible for the activity, a fractionation of cow milk narrowing down on the possible active molecules was performed. Size exclusion chromatography revealed that the fraction of molecules with 60–80 kDa contains the respective activity to lower ID1, ID3 and DLX2 in HSC2 cells (Table 3). We then screened for lactoperoxidase, lactoferrin (both about 80 kDa) and osteopontin (44 kDa) based on their molecular weight and biological activities others than regulation of ID genes^26–28^. By this rather arbitrary approach, we could identify 100 µg/ml lactoperoxidase to decrease basal expression of ID1 and ID3 in HSC2 cells, while 100 µg/ml lactoferrin and 50 ng/ml osteopontin even increased ID1 around 3 to 5-fold (Table 4).

**Table 3:** Expression of ID1, ID3 and DLX2 expression based on size fractionation.

	ID1	ID3	DLX2
Hemoglobin size	12.1 ± 3.8	9.3 ± 1.6	7.6 ± 2.3
Vitamin B12 size	1.85 ± 0.1	2.2 ± 0.3	1.7 ± 0.3

The oral squamous cell carcinoma cell line HSC2 was exposed to a series of fractions with the one peaking at the size of hemoglobin and vitamin B12 shown in this table. Data indicate the x-fold decrease compared to unstimulated control cells. Data represent means and standard deviation of two independent experiments.

**Table 4:** Lactoperoxidase decrease basal expression of ID1 and ID3 in HSC2 cells.

	ID1	ID3	DLX2
Lactoperoxidase	5.0 ± 1.0	4.0 ± 1.0	1.8 ± 0.1
Lactoferrin	0.3 ± 0.08	0.7 ± 0.2	1.0 ± 0.1
Osteopontin	0.2 ± 0.01	0.7 ± 0.03	1.0 ± 0.05

The oral squamous cell carcinoma cell line HSC2 was exposed to 100 µg/ml lactoperoxidase, 100 µg/ml lactoferrin and 50 ng/ml osteopontin for 24 hours followed by gene expression analysis. Data indicate the x-fold decrease compared to unstimulated control cells. Data represent means and standard deviation of two independent experiments.

### MAPK but not TGF-β receptor mediates the changes of ID1, ID3 and DLX2 in HSC2 cells

**Figure 5:**
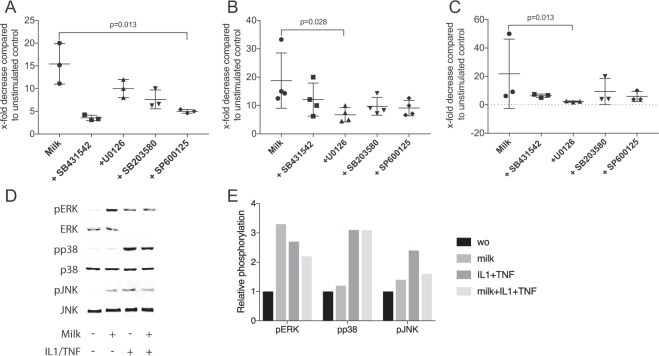
TGF-β receptor and MAPK inhibitors in HSC2 cells exposed to human milk. HSC2 oral squamous cell carcinoma cells were exposed to 5% of aqueous fractions of pasteurized human milk in the presence and absence of SB431542, a TGF-β receptor I kinase inhibitor, and inhibitors against ERK, p38 and JNK signaling, U0126, SB203580, SP600125, respectively. Expression analysis of ID1 (**A**), ID3 (**B**) and DLX2 (**C**) was performed. Data indicate the x-fold decrease compared to unstimulated control cells. (**D**) HSC2 oral squamous cell carcinoma cells were exposed to 5% of aqueous fractions of pasteurized human milk, inflammation provoked by IL1β and TNFα Western blot analysis shows increased phosphorylation signals of particular ERK and to a lesser extent p38 and JNK by exposing HSC2 cells to milk for 30 minutes. (**E**) Data indicate the relative changes normalized to unphosphorylated MAPK and unstimulated control cells. The experiments performed at least three times.

To rule out the involvement of *TGF-β*, the experiments were performed in the presence of SB431542, a TGF-β receptor I kinase inhibitor and recombinant TGF-β^20^. Exposure of HSC2 to SB431542 failed to reverse the activity of human milk to decrease the target genes in HSC2 cells (Fig. 5). In support of these findings, recombinant TGF-β1 had no impact on gene expression (data not shown). However, blocking JNK signaling with SP600125 could partially reverse the milk-mediated decrease of ID1 (Fig. 5A). Blocking ERK signaling by U0126 significantly reversed ID3 and DLX2 expression (Fig. 5B,C). In line with the possible role of MAPK to mediate the effects of human milk, the respective aqueous fraction of milk considerably increased phosphorylation of ERK but not obviously phosphorylation of p38 and JNK (Fig. 5D). The densitometric data are indicated in Supplementary Table 5 and in the Fig. 5E. Therefore, to some extent, MAPK can mediate the effects of milk to decreases ID1, ID3 and DLX2 in HSC2 cells.

## Discussion

Milk and dairy products have been related to head and neck squamous cell carcinoma^4^ and oral cavity and pharyngeal cancer^3^ mainly because of the intrinsic growth factor activities. However, the basal questions how milk affects the genetic signature of oral squamous cell carcinoma has not been answered so far. The present study is a first attempt that describes the regulation of target genes upon exposure of the oral squamous cell carcinoma cell line HSC2 to milk. The main finding was that an aqueous fraction of pasteurized milk, independent of the origin and also upon spray drying in infant formulas, caused substantial expression changes of transcription factors that were identified as negative regulators of RNA polymerase II. A downstream approach confirmed ID1, ID3 and DLX2 to be highly reproducibly down-regulated by milk in the squamous cell carcinoma cell line HSC2.

If we relate the findings to those of others, TGF-β1, being highly abundant in milk, can decrease Id1 in bone marrow mesenchymal cells^29^. TGF-β, however, increases ID1 and ID3 in prostate cancer cells^30^ and Burkitt lymphoma cells^31^. In the present study, SB431542, the TGF-β receptor I kinase inhibitor^32^, failed to reverse the effects of milk on HSC2 and also recombinant TGF-β had no considerable effect. We next tested the role of activin A, known to decrease ID1 and ID3 in keratinocytes^33^ and in the steroidogenic cells of the ovine ovary^25^. Activin A is present in human milk^34^, and activin-like bioactivity is stable against heating^35^. We could, however, not identify any strong changes in IDs upon exposure of HSC2 cells with activin A and also the effects of milk could not be revered by an activin A-neutralizing antibody. Thus, TGF-β1 and activin A do not mediate the effects of milk on target gene expression in HSC2 cells.

We next performed a size fractionation of milk and identified the activity to be present in the large molecular weight fraction. Screening for possible candidates based on their molecular weight and biological activities others than regulation of ID genes^26–28^ allowed us to identify lactoperoxidase purified from bovine milk, but not lactoferrin and osteopontin, to decrease ID1 and ID3 in HSC2 cells. Lactoperoxidase has a molecular weight of approximately 78 kDa^36^. Heat denaturation of lactoperoxidase starts at temperatures from about 70 °C, so pasteurization does not inactivate the enzyme. However, lactoperoxidase is deactivated at pH of 3^36^, and yogurt has around pH of 4. These physico-chemical properties support our findings that fermented milk products are not capable of decreasing the target genes. In the present study, lactoperoxidase, failed to decrease DLX2 expression, but also milk was comparable weak changing this gene. What remains unclear is how lactoperoxidase affects the expression of ID genes? One possible mechanism could be the lowering of reactive oxygen species as, for example, ID3 was identified to be a redox-sensitive gene^37^. Deciphering the role of lactoperoxidase in oral health requires further research.

Future studies should address the molecular mechanism of how lactoperoxidase that is present in various body fluids including tears, saliva and gastric juice, affects gene expression, particular ID1 and ID3, and if this change in transcription factors translates into a cellular response involving proliferation and migration. The viability assay based on formazan formation confirmed the lack of toxicity of 5% milk for HSC2 cells but is not a functional assay. It would be interesting to isolate oral epithelial cells from ID1 and ID3 double knockout mice and perform assays for proliferation and migration in response to milk and lactoperoxidase^38^. We also consider the findings about inhibitors of MAKP signaling to reverse IDs and DLX2 expression as preliminary as the link to lactoperoxidase is missing. Lactoperoxidase was reported to reduce reactive oxygen species and to lower the nuclear factor kappa B (NFkB) signaling in osteoclasts^21^. For example ID1 is upstream of phosphatidylinositol-3-kinase (PI3K)/Akt/NFκB signaling pathway^39^. Thus, the present study raises new questions particular on the role lactoperoxidase and how it regulates ID genes and the respective response in oral epithelial cells.

The clinical relevance remains a matter of speculation. Considering that the ID family function as positive regulators of cell growth it is not surprising that elevated levels of IDs occur in various cancer entreaties including breast, prostate and colon cancer^40^. ID1 overexpression is associated with tumor angiogenesis and poor clinical outcome in oral squamous cell carcinoma^40,41^. Moreover, early dental epithelial markers including DLX2 were differentially overexpressed in ameloblastoma^42^ and DLX2 correlates with the advanced stage of gastric adenocarcinoma^43^. However, it is unclear if *in vivo*, milk and lactoperoxidase decrease ID1, ID3 and DLX2 levels in the healthy and diseased oral mucosa. The research presented here should be a primer for further evaluate the role of milk to change transcription factor expression in oral squamous cell carcinoma. Moreover, this concept could be extended to other malignancies such as the esophagus^44^ and the stomach^45^, which are also exposed to milk. Considering that the effects of milk-derived lactoperoxidase in periodontitis possibly go beyond the antibacterial activity^46,47^, a role of lactoperoxidase in oral health is possible. Finally, since we have not tested milk to regulate genes in nonmalignant cells, this task is to be accomplished in future studies.

## Conclusion

In summary, we show here that milk and lactoperoxidase but not dairy products change gene expression in oral squamous cell carcinoma cells thereby adding a piece of knowledge to our understanding of the possible role of milk in oral oncology.

## Material and Methods

### Ethics, guidelines and regulations

Human milk samples were collected at the Department of Paediatric and Adolescent Medicine, Division of Neonatology, of the Medical University of Vienna after receiving an informed consent and the approval of the ethics committee of the Medical University of Vienna (1021/2017). The informed consent is that “I agree to make my excess milk available to the breast milk bank” but not explicitly for basic research. The ethical board gave permission as all human milk samples were leftovers from a 24-hour pooled sample from the daily meals of the infants. The need for obtaining informed consent from the mothers for the use of left-over breast milk for this study was waived by the ethics committee. If they had not been used for this study, they would have been discarded for other reasons, such as the microbiological risk, due to the milk being more than 72 hours old, it is limited stability and the fact that we could not refreeze leftover milk that had been thawed. Three leftover residual amounts were used for the present study. No mother donated the milk for the study purpose by primary intension. All experiments were performed in accordance with relevant guidelines and regulations.

### Processing of human milk, cow’s milk, infant formula and fermented milk products

Human milk was centrifuged at 20,000 g for 10 min at room temperature. The aqueous fraction was heated at 80 °C for 1 h before freezing. Likewise, three different batches of pasteurized cow’s milk (Billa Bergbauern Heumilch; Spar Halbfett Milch; Hofer Milfina Halbfett Milch) and reconstituted infant formula (Aptamil, Milupa; Babylove, dm; Combiotik, Hipp) were processed. Dairy products yogurt (Ja Natürlich Naturjoghurt; Clever Joghurt; Nöm Naturjoghurt), sour milk (Hofer Milfina Sauermilch; Nöm Sauermilch; Schärdinger Sauermilch), buttermilk (Ja Natürlich Buttermilch Natur; Clever Buttermilch; Spar free from Buttermilch) and whey (Lattella Naturmolke; Clever Fruchtmolke; Spar free from Molke) were similarly subjected to centrifugation to gain the aqueous fraction. The aqueous fractions of each group were pooled and stored at −20 °C until testing. Samples were subjected to not more than two freeze-thaw cycles.

### Cells

The oral squamous cell carcinoma cell line HSC2, originally obtained from Health Science Research Resources Bank (Sennan, Japan), was kindly provided by Prof. Rausch-Fan, Department of Periodontology, Medical University of Vienna, Austria. The oral squamous cell carcinoma cell line TR146 from the European Collection of Authenticated Cell Cultures (ECACC) was kindly provided by Wienfried Neuhaus from the Competence Unit Molecular Diagnostics, Center Health and Bioresources, Austrian Institute of Technology (AIT) GmbH. Human gingival fibroblasts were prepared from explant cultures of three independent donors after approval of the Ethical Committee of the Medical University of Vienna (EK Nr. 631/2007). All cells were cultured in a humidified atmosphere at 37 °C in a growth medium consisting of DMEM, 10% fetal calf serum, and 1% antibiotics (Invitrogen Corporation, Carlsbad, CA, USA).

### Experimental setting

Cells were plated in growth medium at 50,000 cells/cm2 into culture dishes. The following day, cells were exposed to 5% aqueous fractions of human milk, cow’s milk, infant formula and fermented milk products for 24 hours, before gene expression analysis was performed. In indicated experiments, HSC2 cells were exposed to recombinant human TGF-β1 (ProSpec-Tany TechnoGene Ltd., Rehovot, Israel) at 5 ng/ml in the growth medium. SB431542, a TGF-β receptor I kinase inhibitor, was used at 10 µM (Calbiochem, Merck Millipore). In other experiments, 1% of the most prevalent human milk oligosaccharide 2′-Fucosyllactose^48^ (2FL; Jennewein Biotechnologie GmbH, Rheinbreitbach, Germany), or recombinant activina A (100 ng/ml, ProSpec) and an activin A neutralizing antibody (50 ng/ml, ProSpec) was used. HSC2 cells were also expose to 100 µg/ml bovine milk-derived lactoperoxidase, 100 µg/ml bovine milk-derived lactoferrin, and 50 ng/ml recombinant human osteopontin expressed in HEK 293 cells (all Sigma). For MAPK experiments, 10 µM of the inhibitors against ERK (U0126; Cell Signaling Technology, Danvers, MA), p38 (SB 203580, Santa Cruz Biotechnology, Inc., Dallas, TX) and JNK (SP600125, Santa Cruz) were used. Size fractionation of 250 µL aqueous fraction of cow milk was performed with size exclusion chromatography kit using hemoglobin (64.5 kDa) and vitamin B12 (1.4 kDa) serving as internal standard following the recommendations of the manufacturer (Bio-Rad Laboratories, Inc., Hercules, CA).

### Microarray analysis

Total RNA from three replicates was harvested with the RNA Isolation Kit (Extractme, BLIRT S.A., Gdańsk, Poland). The RNA quality of HSC2 cells was determined using the Agilent 2100 Bioanalyzer (Agilent Technologies, Santa Clara, CA, USA). Microarray analysis was performed using the SurePrint G3 Human Gene Expression v2 Microarray (Agilent Technologies, Santa Clara, CA). Array image acquisition was performed with the Agilent G2505B Microarray Scanner and Feature Extraction software version 9.5 (Agilent). Genes that were at least 5-fold regulated in mean were subjected to gene ontology analysis based on the String database (https://string-db.org)^49^.

### Viability assay

For viability experiments, HSC2 was incubated with the aqueous fraction of human milk, cow’s milk, and infant formula at the indicated concentrations. After 24 hours, an MTT (3-[4,5-dimethythiazol-2-yl]-2,5-diphenyltetrazolium bromide; Sigma) solution at a final concentration of 0.5 mg/ml was added to each well of a microtiter plate (CytoOne, Starlab International, Hamburg) for 2 hours at 37 °C. The medium was removed and formazan crystals were solubilized with dimethyl sulfoxide. Optical density was normalized to unstimulated control values.

### qRT-PCR analysis and immunoassay

Reverse transcription was performed with SensiFAST™ cDNA (Bioline, London, UK). The polymerase chain reaction was done with the SensiFAST™ SYBR ROX Kit (Bioline) on a CFX Connect™ Real-Time PCR Detection System (Bio-Rad Laboratories, Hercules, CA). Primer sequences are hID1_F CCAGAACCGCAAGGTGAG, hID1_R GGTCCCTGATGTAGTCGATGA; hID3_F CATCTCCAACGACAAAAGGAG, hID3_R CTTCCGGCAGGAGAGGTT; hDLX2_F AGCAGCTATGACCTGGGCTA, hDLX2_R GCTCAAGGTCCTCCTTCTCA; hGAPDH_F AAGCCACATCGCTCAGACAC, hGAPDH_R GCCCAATACGACCAAATCC. The mRNA levels were calculated by normalizing to the housekeeping gene GAPDH using the ΔΔCt method after exponential expression transformation.

### Western blot

HSC2 cells were exposed to 5% human milk, cow milk and infant formula in growth medium for 72 hours. Cell extracts containing SDS buffer and protease inhibitors (cOmplete™ Protease Inhibitor Cocktail; Sigma, St. Louis, MO) were separated by SDS-PAGE and transferred onto PVDF membranes (Roche Diagnostics GmbH, Mannheim, Germany). For MAPK experiments the cells were serum-starved overnight and exposed to 5% human milk and human IL1β and TNFα (ProSpec-Tany TechnoGene Ltd., Rehovot, Israel) both at 5 ng/ml for 30 minutes. Membranes were blocked and the binding of the first antibody raised against ID1 (B8), ID3 (2B11), DLX2 (B5) and β-actin (C4) (all Santa Cruz Biotechnology) was detected with the appropriate secondary antibody linked to a peroxidase. For MAPK we used first antibody raised against phospho-ERK, phospho-p38 and phospho-JNK from Cell Signaling Technology (Danvers, MA) and to detect the respective non-phosphorylated MAPK, antibodies were from Santa Cruz Biotechnology. Chemiluminescence signals were visualized with the ChemiDoc imaging system (Bio-Rad). For quantification a densitometry based on ImageJ, a free image processing program developed at the National Institutes of Health and the Laboratory for Optical and Computational Instrumentation (LOCI, University of Wisconsin), was performed.

### Statistical analysis

The Kruskal–Wallis test with a post hoc Dunn’s multiple comparisons test was used to assess the difference of the mean rank of each treatment group with the mean rank of the untreated control group. The p-values are indicated in the respective figures.

## Supplementary information

Supplementary Figures

Supplementary Tables
