# Supplementary material for: Milk lactoperoxidase decreases ID1 and ID3 expression in human oral squamous cell carcinoma cell lines
Source: Sci Rep. 2020 Apr 3;10:5836. doi: 10.1038/s41598-020-62390-4 (PMC7125221; doi:10.1038/s41598-020-62390-4)
Supplement: Supplementary file 1 — Supplementary Figures [file 41598_2020_62390_MOESM1_ESM.docx]

**Milk lactoperoxidase decreases ID1 and ID3 expression in human oral squamous cell carcinoma cell lines**

Layla Panahipour^1^, Maria De Biasi^1^, Theresa Sophia Bokor^1^, Alexandra Thajer^2^, Nadja Haiden^3^, Reinhard Gruber*^1,4,5^

1. Department of Oral Biology, Medical University of Vienna, Sensengasse 2a, 1090 Vienna, Austria

2. Department of Paediatrics and Adolescent Medicine, Medical University of Vienna, Währinger Gürtel 18-20, 1090 Vienna, Austria

3. Department of Clinical Pharmacology, Medical University of Vienna, Währinger Gürtel 18-20, 1090 Vienna, Austria

4. Department of Periodontology, School of Dental Medicine, University of Bern, Freiburgstrasse 7, 3010 Bern, Switzerland

5. Austrian Cluster for Tissue Regeneration, Donaueschingenstraße 13, 1200 Vienna, Austria


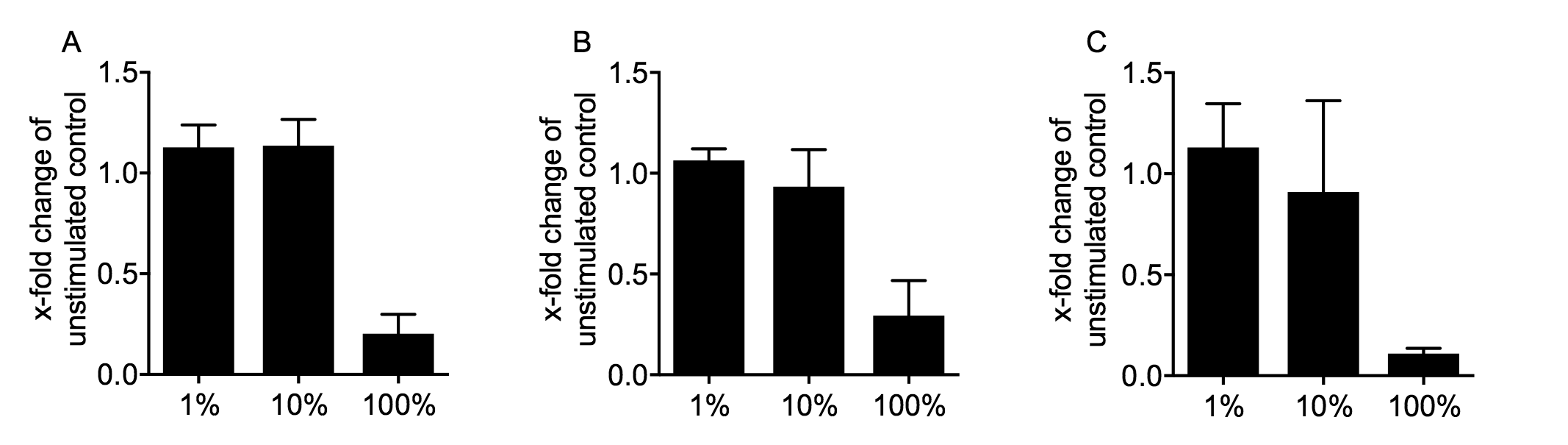


*Supplement Figure 1: Viability tests of HSC2 cells exposed to milk*

HSC2 oral squamous cell carcinoma cells were exposed to the indicated concentrations of aqueous fractions of pasteurized human milk (A), cow’s milk (B), and infant formula (C) for 24 hours, before viability tests with formazan were performed. Data indicate the x-fold change compared to unstimulated control cells.


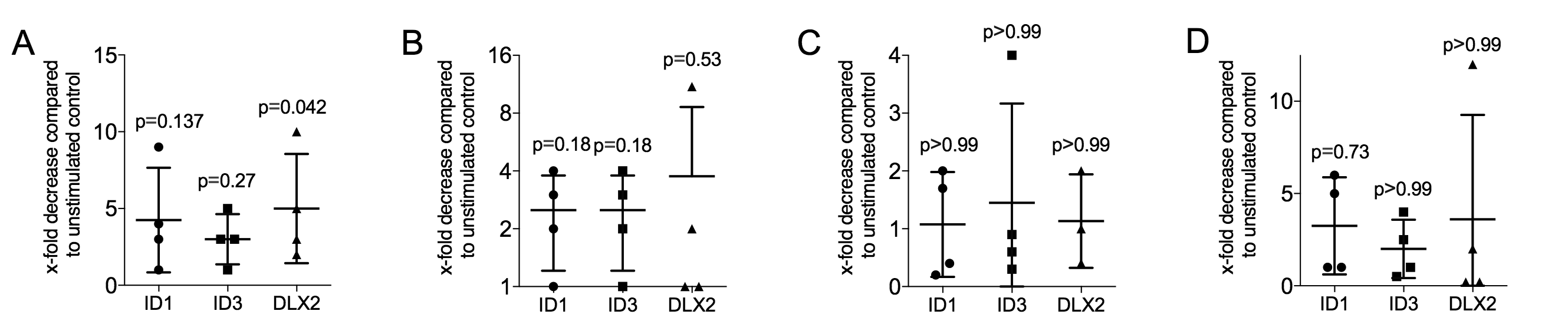


*Supplement Figure 2: ID1, ID3 and DLX2 expression in HSC2 exposed to dairy products*

HSC2 oral squamous cell carcinoma cells were exposed to 5% of aqueous fractions of yoghurt (A), sour milk (B), buttermilk (C), whey (D) for 24 hours, before expression analysis of the target genes ID1, ID3 and DLX2 was performed. Data indicate the x-fold decrease compared to unstimulated control cells.

*
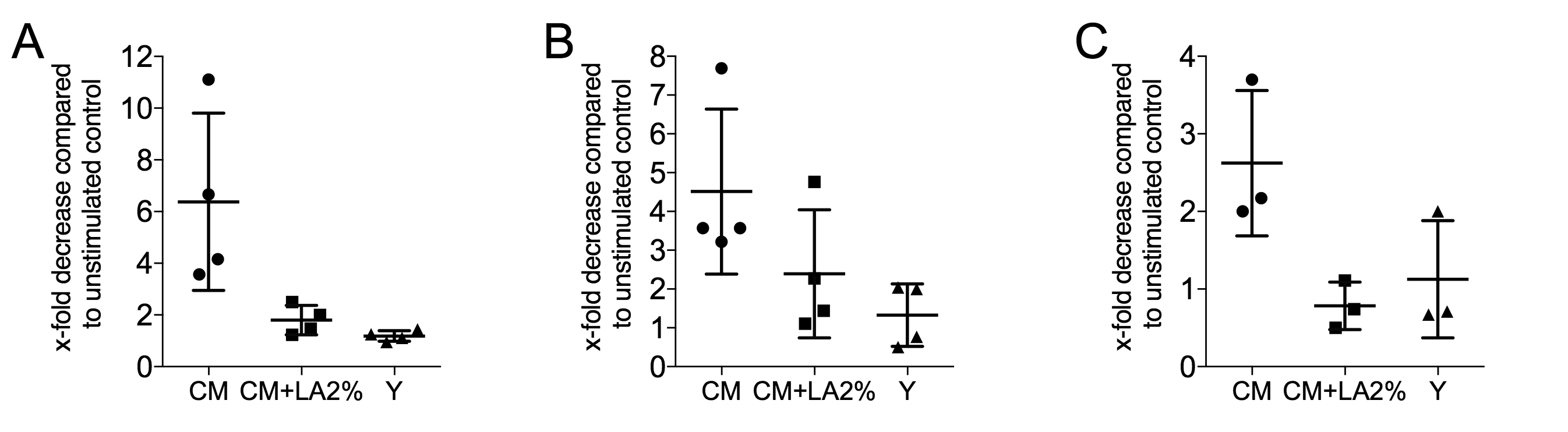
*

*Supplement Figure 3: ID1, ID3 and DLX2 expression in HSC2 exposed to acidified milk*

HSC2 oral squamous cell carcinoma cells were exposed to 5% of aqueous fractions of cow’s milk (A) that was also treated with 2% lactic acid (LA2%) for 18 hours at room temperature (B), or yoghurt (Y; C). Expression analysis of the target genes ID1, ID3 and DLX2 was performed. Data indicate the x-fold decrease compared to unstimulated control cells.


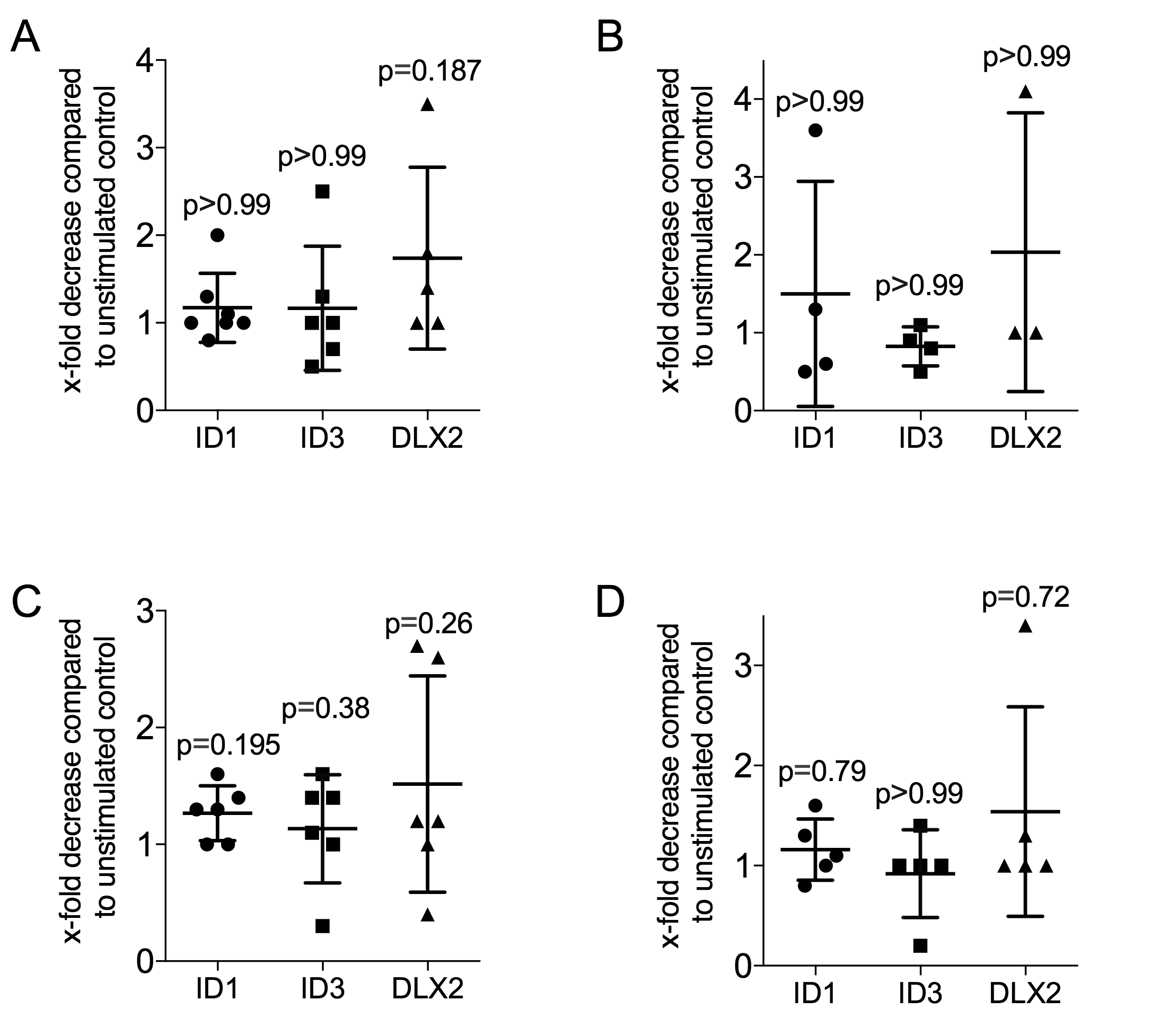


*Supplement Figure 4: ID1, ID3 and DLX2 expression in HSC2 and TR146 exposed to 2FL and 3FL*

HSC2 (A, B) and TR146 (C, D) oral squamous cell carcinoma cells were exposed to 0.2% 2'-Fucosyllactose (2FL, A, C) and 3FL (B, D) for 24 hours, before expression analysis of the target genes ID1, ID3 and DLX2 was performed. Data indicate the x-fold decrease compared to unstimulated control cells.

*Supplement Figure 5: ID1, ID3 and DLX2 expression in HSC2 exposed to activin A (A) and ID1and ID3 expression in HSC2 exposed to human and cow milk compared to combination of milk and antibody (B,C)*

HSC2 oral squamous cell carcinoma cells were exposed to 100ng/ml activin for 24 hours and expression of the target genes ID1, ID3 and DLX2 was performed (A), HSC2 oral squamous cell carcinoma cells were exposed to 5% of aqueous fractions Human and cow milk alone and also antibody 50ng/ml added to milk and target gene expression (ID1, ID3) detected after 24 hours(B,C).


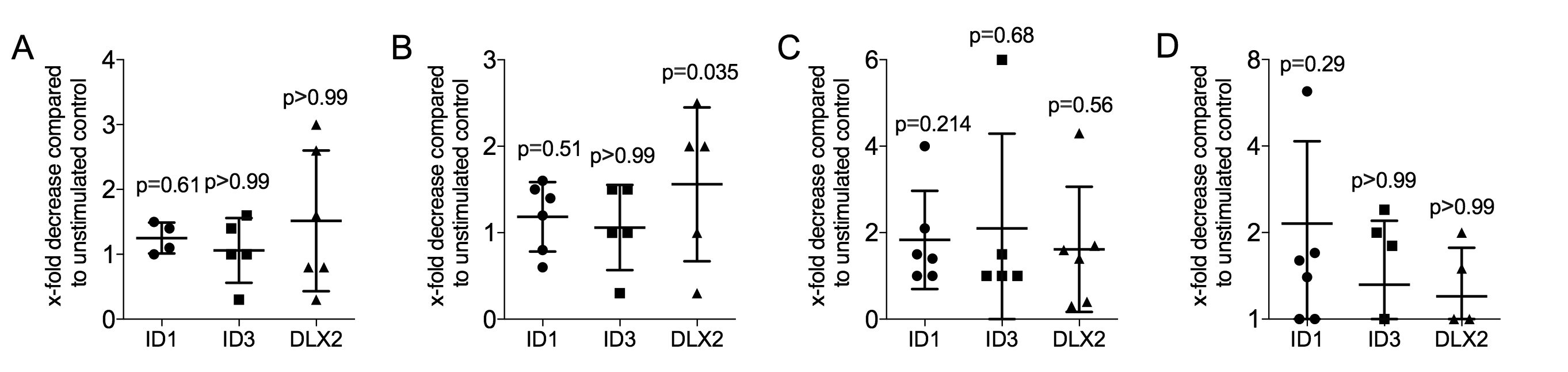


*Supplement Figure 6: ID1, ID3 and DLX2 expression in TR146 exposed to dairy products*

TR146 oral squamous cell carcinoma cells were exposed to 5% of aqueous fractions of yoghurt (A), sour milk (B), buttermilk (C), whey (D) for 24 hours, before expression analysis of the target genes ID1, ID3 and DLX2 was performed. Data indicate the x-fold decrease compared to unstimulated control cells.
