# Supplementary material for: Milk lactoperoxidase decreases ID1 and ID3 expression in human oral squamous cell carcinoma cell lines
Source: Sci Rep. 2020 Apr 3;10:5836. doi: 10.1038/s41598-020-62390-4 (PMC7125221; doi:10.1038/s41598-020-62390-4)
Supplement: Supplementary file 2 — Supplementary Tables [file 41598_2020_62390_MOESM2_ESM.zip › Suppl Tables/Suppl Tables 1, 3, 4, 5/Suppl Table 1, 3, 4, 5.docx]

**Milk lactoperoxidase decreases ID1 and ID3 expression in human oral squamous cell carcinoma cell lines**

Layla Panahipour^1^, Maria De Biasi^1^, Theresa Sophia Bokor^1^, Alexandra Kreissl^2^, Nadja Haiden^3^, Reinhard Gruber*^1,4,5^

1. Department of Oral Biology, Medical University of Vienna, Sensengasse 2a, 1090 Vienna, Austria

2. Department of Paediatrics and Adolescent Medicine, Medical University of Vienna, Währinger Gürtel 18-20, 1090 Vienna, Austria

3. Department of Clinical Pharmacology, Medical University of Vienna, Währinger Gürtel 18-20, 1090 Vienna, Austria

4. Department of Periodontology, School of Dental Medicine, University of Bern, Freiburgstrasse 7, 3010 Bern, Switzerland

5. Austrian Cluster for Tissue Regeneration, Donaueschingenstraße 13, 1200 Vienna, Austria

*Supplement Table 1: Differential expressed genes (>5-fold) in HSC2 cells exposed to 5% aqueous fraction of human milk.*

Probe Set ID Ratio mRna - Description UniGene ID SwissProt mRNA SwissProt accession

17002846 0,04 Homo sapiens dual specificity phosphatase 1 (DUSP1), mRNA. Hs.171695 NM_004417 P28562

16863287 0,07 Homo sapiens FBJ murine osteosarcoma viral oncogene homolog B (FOSB), transcript variant 2, mRNA. Hs.590958 NM_001114171 P53539

17082083 0,07 Homo sapiens v-maf avian musculoaponeurotic fibrosarcoma oncogene homolog A (MAFA), mRNA. Hs.521914 NM_201589 Q8NHW3

16683377 0,09 Homo sapiens inhibitor of DNA binding 3, dominant negative helix-loop-helix protein (ID3), mRNA. Hs.76884 NM_002167 Q02535

16962661 0,11 Homo sapiens claudin 1 (CLDN1), mRNA. Hs.439060 NM_021101 O95832

16951797 0,12 Homo sapiens eomesodermin (EOMES), transcript variant 1, mRNA. Hs.591663 NM_001278182 O95936

17076861 0,13 Homo sapiens CCAAT/enhancer binding protein (C/EBP), delta (CEBPD), mRNA. Hs.440829 NM_005195 P49716

17100420 0,14 Homo sapiens NOTCH-regulated ankyrin repeat protein (NRARP), mRNA. Hs.732282 NM_001004354 Q7Z6K4

16774130 0,15 Homo sapiens FRAS1 related extracellular matrix protein 2 (FREM2), mRNA. Hs.253994 NM_207361 Q5SZK8

16802232 0,15 Homo sapiens SMAD family member 6 (SMAD6), transcript variant 3, non-coding RNA. Hs.153863 NR_027654 O43541

16666738 0,15 Homo sapiens cysteine-rich, angiogenic inducer, 61 (CYR61), mRNA. Hs.8867 NM_001554 O00622

16697370 0,15 Homo sapiens prostaglandin-endoperoxide synthase 2 (PTGS2), mRNA. Hs.196384 NM_000963 P35354

16705011 0,15 Homo sapiens dickkopf WNT signaling pathway inhibitor 1 (DKK1), mRNA. Hs.40499 NM_012242 O94907

16967327 0,16 Homo sapiens transmembrane protease, serine 11E (TMPRSS11E), mRNA. Hs.201877 NM_014058 Q9UL52

16677278 0,16 Homo sapiens activating transcription factor 3 (ATF3), transcript variant 3, mRNA. Hs.460 NM_001030287 P18847

16716478 0,17 Homo sapiens ankyrin repeat domain 1 (cardiac muscle) (ANKRD1), mRNA. Hs.448589 NM_014391 Q15327

16967771 0,17 Homo sapiens chemokine (C-X-C motif) ligand 8 (CXCL8), mRNA. Hs.624 NM_000584 P10145

16657534 0,17 Homo sapiens sterile alpha motif domain containing 11 (SAMD11), mRNA. Hs.335293 NM_152486 Q96NU1

16903491 0,17 Homo sapiens Rho family GTPase 3 (RND3), transcript variant 1, mRNA. Hs.6838 NM_001254738 P61587

16706727 0,18 Homo sapiens chromosome 10 open reading frame 99 (C10orf99), mRNA. Hs.298713 NM_207373 Q6UWK7

16990848 0,18 Homo sapiens adrenoceptor beta 2, surface (ADRB2), mRNA. Hs.2551 NM_000024 P07550

16689042 0,18 Homo sapiens synapse defective 1, Rho GTPase, homolog 2 (C. elegans) (SYDE2), mRNA. Hs.670497 NM_032184 Q5VT97

16911261 0,18 Homo sapiens bone morphogenetic protein 2 (BMP2), mRNA. Hs.73853 NM_001200 P12643

16870384 0,19 Homo sapiens jun D proto-oncogene (JUND), transcript variant 1, mRNA. Hs.2780 NM_001286968 P17535

17004903 0,19 Homo sapiens endothelin 1 (EDN1), transcript variant 2, mRNA. Hs.511899 // Hs.713645 NM_001168319 P05305

17004208 0,18 Homo sapiens forkhead box C1 (FOXC1), mRNA. Hs.348883 NM_001453 Q12948

16687875 0,19 Homo sapiens jun proto-oncogene (JUN), mRNA. Hs.696684 NM_002228 P05412

17004194 0,19 Homo sapiens forkhead box Q1 (FOXQ1), mRNA. Hs.591352 NM_033260 Q9C009

17034172 6,51 Homo sapiens corneodesmosin (CDSN), mRNA. Hs.310958 // Hs.556031 NM_001264 Q15517

17005582 0,20 Homo sapiens histone 1, H2bg, mRNA (cDNA clone IMAGE:6668499), partial cds. Hs.182137 BC056264 P62807

16905108 0,20 GRCh38:2:172099439:172102900:-1 gene:ENSG00000115844 gene_biotype: protein_coding Hs.419 ENST00000234198 Q07687

16725871 0,20 Homo sapiens secretoglobin, family 1A, member 1 (uteroglobin) (SCGB1A1), mRNA. Hs.523732 NM_003357 P11684

16693249 0,20 Homo sapiens thioesterase superfamily member 5 (THEM5), mRNA. Hs.132648 NM_182578 Q8N1Q8

16666799 0,20 Homo sapiens chloride channel accessory 4 (CLCA4), transcript variant 1, mRNA. Hs.567422 NM_012128 Q14CN2

16748989 0,20 Homo sapiens phosphodiesterase 3A, cGMP-inhibited (PDE3A), transcript variant 1, mRNA. Hs.386791 // Hs.737522 NM_000921 Q14432

16962584 0,21 Homo sapiens B-cell CLL/lymphoma 6 (BCL6), transcript variant 1, mRNA. Hs.478588 NM_001706 P41182

16861997 0,21 Homo sapiens ZFP36 ring finger protein (ZFP36), mRNA. Hs.534052 NM_003407 P26651

16792181 0,21 Homo sapiens forkhead box A1 (FOXA1), mRNA. Hs.163484 NM_004496 P55317

16912362 0,21 Homo sapiens inhibitor of DNA binding 1, (ID1), transcript variant 1, mRNA. Hs.504609 NM_002165 P41134

17016390 0,28 Homo sapiens histone cluster 1, H2bg (HIST1H2BG), mRNA. Hs.591809 NM_003518 P62807

16682098 0,22 PREDICTED: Homo sapiens EPH receptor A2 (EPHA2), transcript variant X1, mRNA. Hs.171596 XM_005245751 P29317

16825371 0,22 Homo sapiens nuclear protein, transcriptional regulator, 1 (NUPR1), transcript variant 1, mRNA. Hs.513463 NM_001042483 O60356

16774053 0,22 Homo sapiens cyclin A1 (CCNA1), transcript variant 2, mRNA. Hs.417050 NM_001111045 P78396

17050455 0,22 Homo sapiens forkhead box P2 (FOXP2), transcript variant 5, mRNA. Hs.282787 NM_001172766 O15409

16958844 0,22 Homo sapiens GATA binding protein 2 (GATA2), transcript variant 1, mRNA. Hs.367725 NM_001145661 P23769

16859205 0,23 Homo sapiens urothelial cancer associated 1 (non-protein coding) (UCA1), long non-coding RNA. Hs.644234 NR_015379 Q2VCE5

16722987 0,23 Homo sapiens leucine zipper protein 2 (LUZP2), transcript variant 1, mRNA. Hs.144138 // Hs.655032 NM_001009909 Q86TE4

16696824 0,23 Homo sapiens ABL proto-oncogene 2 (ABL2), transcript variant d, mRNA. Hs.159472 NM_001136000 P42684

16903897 0,24 Homo sapiens nuclear receptor subfamily 4, group A, member 2 (NR4A2), mRNA. Hs.563344 uc002tyx.4 P43354

16987395 0,24 Homo sapiens Rho-related BTB domain containing 3 (RHOBTB3), mRNA. Hs.445030 NM_014899 O94955

17067890 0,24 GRCh38:8:37695751:37700021:1 gene:ENSG00000183779 protein_coding Hs.744591 ENST00000331569 Q9H7S9

16761269 0,24 Homo sapiens C-type lectin domain family 7, member A (CLEC7A), transcript variant 2, mRNA. Hs.143929 NM_022570 Q9BXN2

16699533 0,24 Homo sapiens dual specificity phosphatase 10 (DUSP10), transcript variant 3, non-coding RNA. Hs.497822 NR_111940 Q9Y6W6

17024079 0,25 Homo sapiens mitogen-activated protein kinase kinase kinase 5 (MAP3K5), mRNA. Hs.186486 NM_005923 Q99683

17080516 0,25 Homo sapiens ectonucleotide pyrophosphatase/phosphodiesterase 2 (ENPP2), TV 2, mRNA. Hs.190977 NM_001040092 Q13822

17044253 0,25 Homo sapiens glycoprotein (transmembrane) nmb (GPNMB), transcript variant 1, mRNA. Hs.190495 NM_001005340 Q14956

16681304 0,25 GRCh38:1:8013427:8015633:-1 gene:ENSG00000116285 protein_coding Hs.605445 // Hs.713029 ENST00000467067 K7EJB4

17121236 5,02 PREDICTED: Homo sapiens uncharacterized LOC100128108 (LOC100128108), TV X3, mRNA. Hs.104864 // Hs.187134 XM_006720814 H0YN89

16819207 5,35 GRCh38:16:56608199:56609497:1 gene:ENSG00000125148 protein_coding Hs.534330 ENST00000245185 P02795

16689384 5,60 Homo sapiens guanylate binding protein 4 (GBP4), mRNA. Hs.409925 NM_052941 Q96PP9

16914171 5,73 Homo sapiens peptidase inhibitor 3, skin-derived (PI3), mRNA. Hs.112341 NM_002638 P19957

16707180 6,10 Homo sapiens interferon-induced protein with tetratricopeptide repeats 2 (IFIT2), mRNA. Hs.437609 NM_001547 P09913

16977058 6,14 Homo sapiens chemokine (C-X-C motif) ligand 11 (CXCL11), mRNA. Hs.632592 NM_005409 O14625

16984010 6,17 Homo sapiens interleukin 7 receptor (IL7R), transcript variant 1, mRNA. Hs.591742 NM_002185 P16871

16693335 6,38 Homo sapiens late cornified envelope 3D (LCE3D), mRNA. Hs.244349 NM_032563 Q9BYE3

17031720 6,51 Homo sapiens corneodesmosin (CDSN), mRNA. Hs.310958 // Hs.556031 NM_001264 Q15517

16838418 6,60 Homo sapiens C1q and TNF related protein 1 (C1QTNF1), transcript variant 1, mRNA. Hs.201398 NM_030968 Q9BXJ1

16771417 7,21 Homo sapiens 2-5-oligoadenylate synthetase-like (OASL), transcript variant 3, mRNA. Hs.118633 NM_001261825 Q15646

16977052 7,42 Homo sapiens chemokine (C-X-C motif) ligand 10 (CXCL10), mRNA. Hs.632586 NM_001565 P02778

17121848 7,83 Homo sapiens dynein, axonemal, heavy chain 17 (DNAH17), mRNA. Hs.375975 NM_173628 Q9UFH2

16872777 8,13 Homo sapiens cornifelin (CNFN), mRNA. Hs.148590 NM_032488 Q9BYD5

16669796 9,05 Homo sapiens thioredoxin interacting protein (TXNIP), mRNA. Hs.709057 NM_006472 Q9H3M7

16671104 10,88 Homo sapiens small proline-rich protein 1A (SPRR1A), transcript variant 1, mRNA. Hs.46320 NM_001199828 P35321

16743721 13,09 Homo sapiens matrix metallopeptidase 1 (interstitial collagenase) (MMP1), transcript variant 2, mRNA. Hs.83169 NM_001145938 P03956

16743707 20,18 Homo sapiens matrix metallopeptidase 10 (stromelysin 2) (MMP10), mRNA. Hs.2258 NM_002425 P09238

16914395 24,11 Homo sapiens matrix metallopeptidase 9 (MMP9), mRNA. Hs.297413 NM_004994 P14780

Supplement Table 3: Primers for screening apart from ID1, ID3 and DLX5 (data are x-fold change when HSC2 cells were exposed to 5% human milk (HMM) and cow milk (CM).

Gene ID x-fold (HMM) x-fold (CM) Forward Reverse

hBMP2 0.37±0.25 0.83±0.47 cagaccaccggttggaga ccactcgtttctggtagttcttc

hCEBPD 0.16±0.04 2.07±1.88 ggacataggagcgcaaagaa gcttctctcgcagtttagtgg

hCLDN1 0.23±0.08 0.63±0.49 ttgactccttgctgaatctgag ggccacaaagattgctatcac

hCDSN_F 0.96±0.43 0.72±0.51 atgatggcactgctgctg aaggtgccaatgctcttagc

hCXCL10 1.13±0.71 0.70±0.50 gaaagcagttagcaaggaaaggt gacatatactccatgtagggaagtga

hCXCL11 1.30±1.38 0.47±0.23 agtgtgaagggcatggcta tcttttgaacatggggaagc

hCYR61 0.57±0.15 1.15±0.78 aaacccggatttgtgaggt gctgcatttcttgcccttt

hDKK1 0.86±0.38 0.51±0.45 caggcgtgcaaatctgtct aatgattttgatcagaagacacacata

hDUSP1 0.37±0.03 1.14±0.80 cgaggccattgacttcataga ctggcagtggacaaacacc

hFOSB 1.34±1.52 4.26±3.92 tgtcttcggtggactccttc gaaggaaccgggcatttc

hFOXC1 0.26±0.04 0.56±0.33 tgaacgggaatagtagctgtca ggacgtgcggtacagagac

hJUN 2.03±2.21 1.61±1.00 ccaaaggatagtgcgatgttt ctgtccctctccactgcaac

hJUND 1.77±1.24 2.79±1.51 aagtcctcagccacgtcaac gccatgcccgactcagta

hLAMC2 0.61±0.22 0.46±0.30 cagaagcccagaaggttgat acactgagaggctggtccat

hLOC105377957 4.22±3.78 0.59±0.32 cttggccaacagtgactgac ccctgtgaggaggactcaac

hMAFA 1.22±1.07 0.87±0.44 aacggacaatcaggaaatgag gtttagaggtgatccgtcttcc

hOASL_Tv1&2 1.35±0.57 0.25±0.18 ttgtgtcagaaaacagctcaaaa gcaacgatgtcccatctgta

hPI3 5.48±6.56 1.00±1.21 gtggtggtgttcctcatcg acggcctttgacagtgtctt

hPLEK2 2.65±2.16 0.87±0.61 gagagtgacccctcccaag ttgagtcttcagcttaatgaggag

hPTGS2 2.46±1.62 0.57±0.35 cttcacgcatcagtttttcaag tcaccgtaaatatgatttaagtccac

hSerpine 0.80±0.08 1.06±0.46 aaggcacctctgagaacttca cccaggactaggcaggtg

hSPRR1A 1.60±0.85 1.23±0.42 tcgggtgcatttgaggat aaggaagactagggatggttca

hTXNIPv1 1.56±0.63 1.27±1.39 acgcttcttctggaagacca aagctcaaagccgaacttgt

hZFP36 2.05±0.69 1.11±1.15 cccaagtgtgcaagctcag ccccaagaacctcggaag

Supplement Table 4: Protein expression analysis of HSC2 exposed to milk based on Western blot analysis. Thus, aqueous fraction of pasteurized human milk, cow milk and infant formula lower the protein levels of ID1 and ID3 in HSC2 cells.

wo MM CM IF

ID1 117.919 73.326 58.376 18.364

ID3 217.376 103.348 131.992 130.587

DLX2 142.234 96.829 89.213 122.265

Actin 116.264 112.689 113.075 118.412

Supplement Table 5: Milk increases phosphorylation of ERK in HSC2 cells. The aqueous fraction of milk considerably increased phosphorylation of ERK but has no strong effect on changing the phosphorylation of p38 and JNK, also in the presence of inflammatory cytokines.

wo milk IL1+TNF milk+IL1+TNF

pERK 67.813 269.389 207.213 195.509

ERK 213.979 270.945 252.361 293.549

pp38 101.178 130.611 363.826 307.893

p38 262.691 278.669 290.123 247.907

pJNK 103.790 167.442 232.280 153.227

JNK 338.390 386.148 318.800 326.561
